# Effect of coarctation of aorta anatomy and balloon profile on the outcome of balloon angioplasty in infantile coarctation

**DOI:** 10.1186/s12872-021-02396-6

**Published:** 2021-12-15

**Authors:** Hamid Amoozgar, Narjes Nouri, Sajad Shabanpourhaghighi, Neda Bagherian, Nima Mehdizadegan, Mohammad Reza Edraki, Amir Naghshzan, Hamid Mohammadi, Gholamhossein Ajami, Ashkan Abdollahi

**Affiliations:** 1 Neonatal Research Center, School of Medicine, Shiraz University of Medical Sciences, Shiraz, Iran; 2 Pediatric Department, School of Medicine, Namazi Hospital, Shiraz University of Medical Sciences, Shiraz, Iran; 3 School of Medicine, Shiraz University of Medical Sciences, Jahrom, Iran; 4 The Cardiovascular Research Center, Shiraz University of Medical Sciences, Shiraz, Iran; 5 Student Research Committee, Shiraz University of Medical Sciences, Shiraz, Iran

**Keywords:** Aortic coarctation, Angioplasty, Balloon, Infant, Recoarctation

## Abstract

**Objective:**

Coarctation of the Aorta (CoA) is a relatively common cardiovascular disorder. The present study aimed to evaluate the effect of COA anatomy and high versus low-pressure balloons on the outcome of balloon angioplasty among neonates and infants.

**Methods:**

In this retrospective study, the neonates and infants undergoing balloon angioplasty at Namazi hospital were enrolled. After balloon angioplasty, immediate data results were promptly recorded.Moreover, midterm echocardiographic information was collected via electronic cardiac records of pediatric wards and clinical and echocardiographic data at least 12 months after balloon angioplasty. Finally, data were analyzed using SPSS-20.

**Results:**

In this study, 42 infants were included. The median age at the time of balloon angioplasty was 1.55 (range 0.1–12) months and 66.7% of the patients were male. The mean pressure gradient of coarctation was 38.49 ± 24.97 mmHg, which decreased to 7.61 ± 8.00 mmHg (P < 0.001). A high-pressure balloon was used in 27, and a low-pressure balloon was used in 15 patients. COA's pressure gradient changed 30.89 ± 18.06 in the high-pressure group and 24.53 ± 20.79 in the low-pressure balloon group (P = 0.282). In the high-pressure balloon group, 14.81% and in the low-pressure group, 33.33% had recoarctation and need second balloon angioplasty (p < 0.021). The infant with discrete coarctation had a higher decrease in gradient and lower recoarctation.

**Conclusion:**

Recoarctation rate was lower in the high-pressure balloon. The infant with discrete COA had a better response to the balloon with more decrease in gradient and lower recoarctation rate. Therefore, the stenotic segment anatomy needs to be considered in the selection of treatment methods.

## Introduction

Coarctation of the aorta (CoA) is the fifth common congenital heart defect and includes almost 6–8% of all congenital heart diseases, which is estimated to occur one in every 2500 births [1, 2]. Like other left-sided obstructive heart defects, CoA is more common among boys(male to female ratio = 1.23 to 1.74) [3, 4]. The clinical manifestations of CoA vary from an infant with heart failure to asymptomatic systemic hypertension or murmur in children and adults who are accidentally diagnosed in clinical examinations [1].

If there is no action to correct CoA, most patients will die during infancy, but in survival cases, it will be associated with cardiac morbidity and mortality. In the largest study on the natural course of the disease by Campbell et al., a mean lifetime of 34 years was reported for patients with CoA if they survived the first year of life. The leading causes of death were heart failure (25%), aortic dissection (21%), bacterial endarteritis (18%), and intracranial hemorrhage (ICH) (12%), respectively [5]. Treatment involves thoracotomy and correction of stenosis using various techniques, the most widely used, including excision of the stenosis area and end-to-end anastomosis, balloon angioplasty, and percutaneous stenting [1].

Surgery was the only option for CoA treatment during four decades. Thereafter, balloon angioplasty was introduced as a less invasive procedure in 1982 [6]. Balloon angioplasty was performed through the femoral artery or femoral vein, and also cutting balloon was used for angioplasty [7]. As it was mentioned, the mortality rate is remarkable in the surgical repair of COA. However, surgery compared to native CoA surgery is challenging in recoarctation and is associated with a high mortality and morbidity rate, particularly in neonates. In such patients, the less invasive balloon angioplasty is the preferred technique [8, 9]. Some studies have not considered a ideal technique for early treatment of CoA because of considerable residual or recoarctation rates (about 27%), organ ischemia, and aneurysm formation compared to surgical procedures, particularly in infancy [8–10]. On the other hand, midterm results of this technique including recoarctation and aneurysm have been reported to be better and comparable with surgical methods in elder children, adolescents, and adults than in neonates [8, 11].

The most common complication second to balloon angioplasty is an injury to the femoral artery, which is common in children < 1 year of age. However, its prevalence has decreased since the introduction of the low-profile balloon [11]. Less common complications include femoral artery bleeding and cerebrovascular events (CVA). Paradoxical hypertension is rare in balloon angioplasty [12]. Overall, aortic rupture, aneurysm formation, recoarctation, and death are likely to occur in both methods [13]. Each method has some advantages and disadvantages, particularly in infants. Moreover, due to the duration of the follow-up period and the low numbers of patients in different studies, it is impossible to prioritize each method accurately [14, 15].

The present study aimed to evaluate the immediate and midterm results of CoA balloon angioplasty among infants with native CoA according to the COA segment's anatomy and tocompare the outcome of low-pressure and high-pressure balloons.

## Methods

In this retrospective study, all infants undergoing balloon angioplasty at Namazi hospital affiliated with Shiraz University of Medical Sciences, Shiraz, Iran, from January 2015 to January 2018 were enrolled. Samples were selected by convenient and available sampling method. Inclusion criteria were neonates and infants (< 1 year of age) who had electronic records and followed-up at least for one year after balloon angioplasty and had a discrete stenosis segment. But the patients with ages > 1 year, no access to follow-up results, COA associated with complex intracardiac congenital heart disease, and long segment hypoplastic aortic arch without discrete stenosis not suitable for balloon angioplasty were excluded.

Data of immediate results of balloon angioplasty were recorded from catheterization charts. Also, midterm echocardiographic information was collected from an electronic pediatric cardiac record at least 12 months after balloon angioplasty. Additional information such as age, type of balloon, size of the balloon, ascending aortic pressure, descending aortic pressure, COA gradient, response to balloon dilation, ascending aortic diameter, descending aortic diameter, stenotic site diameter, and complications were also recorded.

**Fig. 1:**
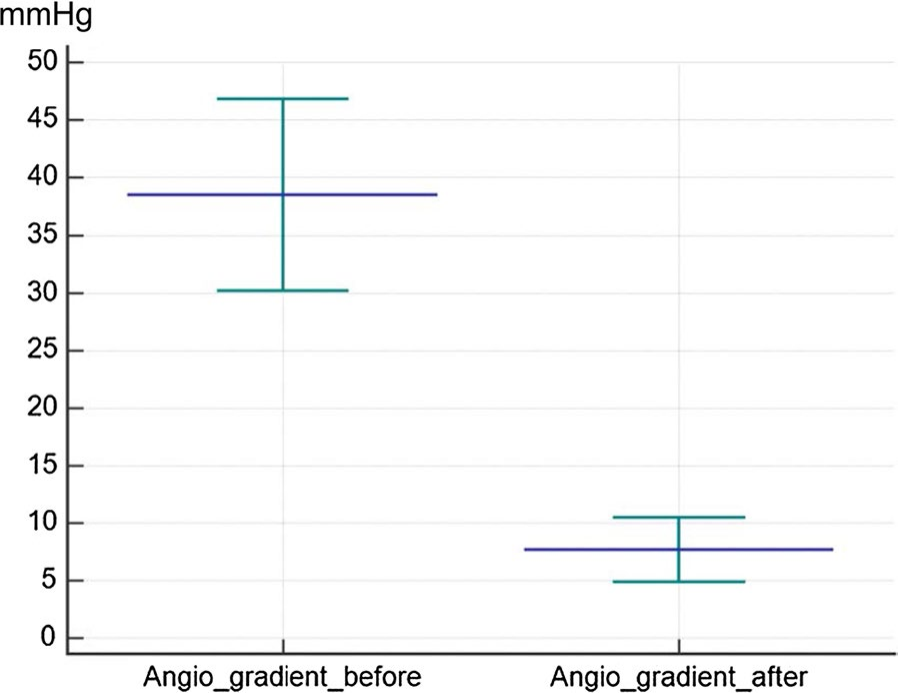
Pressure gradient before and after balloon angioplasty of coarctation

According to the anatomy of the aortic arch, the patients were grouped to discrete coarctation (normal z score of the proximal ascending aorta, distal ascending aorta, aortic isthmus, and descending aorta,) (group 1), hypoplastic aortic isthmus (Z score of aortic isthmus less than -2) in association with discrete narrowing (group 2), and aortic arch hypoplasia, was defined as an aortic arch diameter less than 50% of the ascending (Z score of the distal ascending aorta, aortic isthmus less than − 2 associated with discrete narrowing (group 3) (Fig. 1).
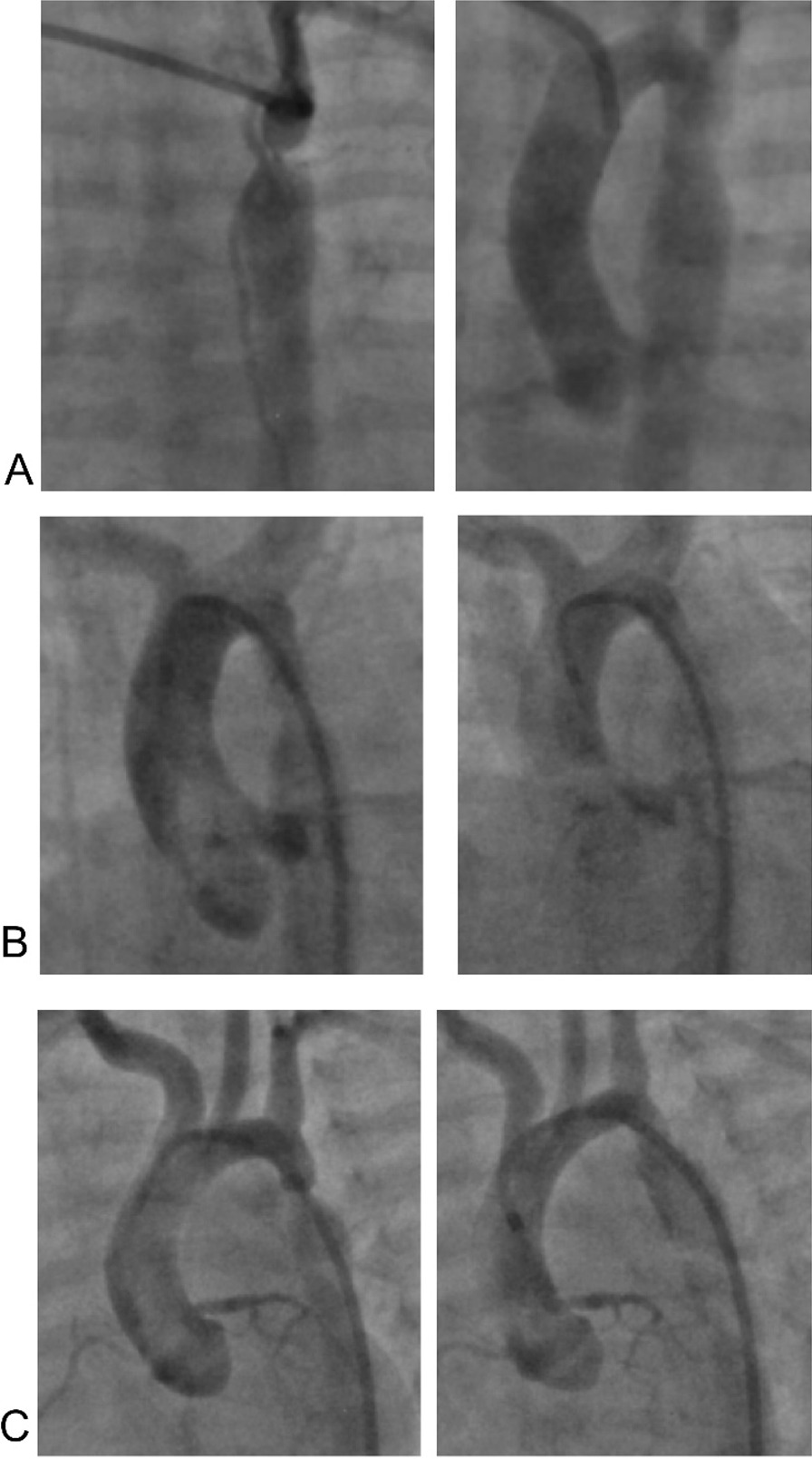

Data were analyzed by the Statistical Package for the Social Sciences (SPSS) software (Version 20). The Mann–Whitney U test and Chi-square were used to compare groups and outcomes, respectively. Wilcoxon test was used to evaluate the related samples. Person correlation was used to evaluate the correlation. P less than 0.05 was considered statistically significant.

## Results

In this study, 42 patients were included. The median age at the time of balloon angioplasty was 1.55 (range 0.1–12) months, and 66.66% were male. In 42.85% of patients, COA was associated with noncomplex chronic heart diseases (CHD) such as the bicuspid aortic valve, mild aortic stenosis, small patent ductus arteriosus, mild mitral stenosis, and small ventricular septal defect (VSD). Six patients had severe heart failure and acidosis and needed starting prostaglandin E1 before the procedure, and other infants had clinically compensated heart failure with dilated left or right ventricle and pulmonary hypertension that needed urgent intervention. The mean diameter and length of the angioplasty balloon were 7.01 ± 1.47 mm and 37.07 ± 11.40 mm.

Before the balloon angioplasty, mean ascending and descending aortic systolic pressures were 107.61 ± 32.71 mmHg and 69.21 ± 20.78 mmHg, respectively; with a mean pressure gradient of 38.49 ± 24.97 mmHg that decreased to 7.61 ± 8.00 mmHg (P < 0.001) (Fig. 1). In addition, the mean days of admission were 9.72 ± 4.02 days.

Out of 42 infants, nine patients needed reintervention at the mean age of 7.57 ± 3.10 months. Table 1 compares two groups of the infant who need reintervention and did not need reintervention.

**Table 1:** Comparison between the infant who needs reintervention and did not reintervention in fist balloon angioplasty of coarctation of the aorta

Variables	Mean ± SD		p value
	Non reintervention (n = 33)	Reintervention (n = 9)	
Age (month)	2.46 ± 2.89	2.57 ± 3.10	0.030
Balloon/coarctation ratio	3.52 ± 0.79	3.16 ± 0.61	0.257
Descending thoracic aorta dimension (mm)	6.23 ± 1.84	5.71 ± 1.71	0.154
Preprocedural coarctation diameter (mm)	1.98 ± 0.82	1.68 ± 0.78	0.146
Preprocedural gradient (mm hg)	38.49 ± 24.97	33.71 ± 23.12	0.643
Postprocedural coarctation diameter (mm)	5.17 ± 1.72	4.89 ± 1.88	0.471
Post procedural gradient (mm hg)	6.91 ± 6.20	8.33 ± 4.68	0.296
z-value of the transverse arch	− 2.54 ± 1.45	− 3.12 ± 2.34	0.257

In this study, nine patients (21.42%) with a mean age of 7.82 ± 3.33 months who were mostly boys (77.81%), required second balloon angioplasty. Mean angioplasty balloon length and diameter were 9.11 ± 1.54 and 36.67 ± 5.32, respectively. Before the second balloon angioplasty, the mean gradient was 44.47 ± 34.14 mmHg that decreased to 9.02 ± 4.14 mmHg after angioplasty (P = 0.022). One patient underwent a third balloon angioplasty during the study (Table 2).

**Table 2:** Demographic information and first CoA balloon angioplasty in infants with native CoA

Variable	Mean ± SD			p value
	< 1 mo (n = 8)	1–3 mo (n = 9)	> 3 mo (n = 25)	
Age (month)	0.42 ± 0.32	1.99 ± 0.66	16.10 ± 9.27	–
Balloon diameter (mm)	6.54 ± 0.96	7.16 ± 1.26	7.2 ± 3.11	0.062
Pressure gradient before balloon (mm Hg)	28.38 ± 10.17	38.21 ± 13.71	41.99 ± 15.11	0.213
Pressure gradient after balloon (mm Hg)	8.42 ± 4.21	7.91 ± 3.79	9.81 ± 5.64	0.125
Recoarctation rate number (%)	4 (50)	3 (33.3)	2 (8)	0.035
Admission duration (day)	15.07 ± 12.64	7.95 ± 9.96	2.67 ± 1.15	0.539

### Effect of high-pressure balloon

A high-pressure balloon was used in 27, and a low-pressure balloon was used in 15 patients. COA's pressure gradient changed 30.89 ± 18.06 in the high-pressure group and 24.53 ± 20.79 in the low-pressure balloon group (P = 0.038).

**Fig. 2:**
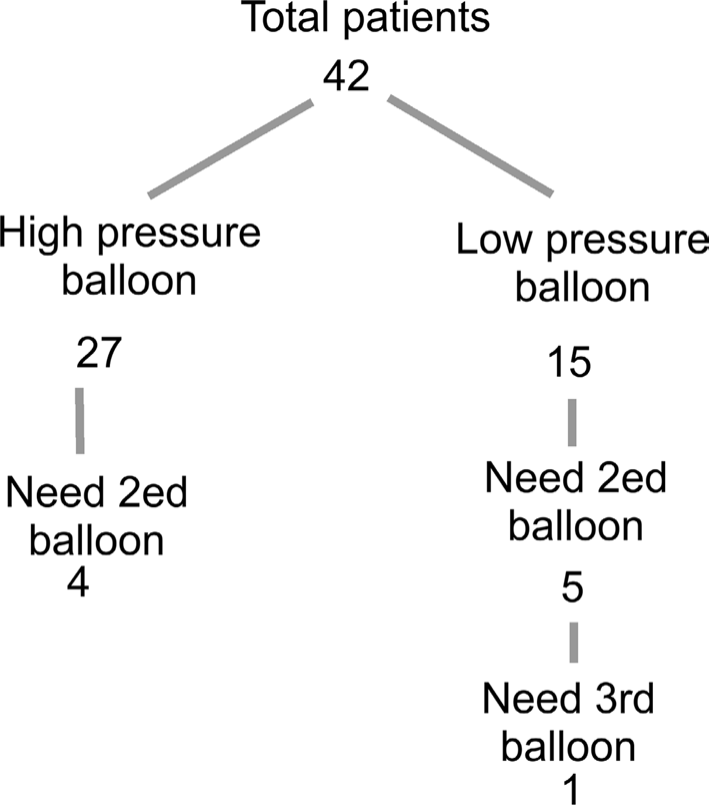
The outcomes of using high pressure versus low-pressure balloon

Out of 27 patients who had high-pressure balloon angioplasty four patients (14.81%) had recoarctation and needed second balloon angioplasty, and out of 15 patients, who had low-pressure balloon angioplasty, five (33.33%) patients had recoarctation and needed second balloon angioplasty (p < 0.021). One patient in the low-pressure group needed third balloon angioplasty, but no patient in the high-pressure group needed a third balloon (Fig. 2).

### Effect of aortic arch anatomy

Out of 42 patients, 19 patients had discrete coarctation, 12 patients had hypoplastic isthmus with discrete stenosis, and 11 had a hypoplastic aortic arch with discrete stenosis.

The mean decrease of the gradient was 40.23 ± 10.62, 32.19 ± 20.12, and 31.75 ± 19.82 in group 1, group 2, and group 3, respectively (P = 0.037).

Recoarctation was in one patient (5.26%) in group 1, three patients (25.00%) in group 2, and five patients (45.45%) in group 3.

In the follow-up, three cases of aneurysm formation at the site of angioplasty. One aneurysm was with a low-pressure balloon, and two aneurysms were with a high-pressure balloon. All the aneurysms were in group 2 of aortic arch anatomy. Two patients had a loss of pulses for 24 h after the balloon angioplasty that improved by heparin infusion.

There was no statistically significant correlation between balloon diameter and change of gradient (P = 0.75).

## Discussion

This study aimed to evaluate the effect of coarctation of aorta anatomy and balloon profile on the outcome of balloon angioplasty in infantile coarctation.

Recoarctation was lower in infants with discrete coarctation anatomy without arch or isthmus hypoplasia. Therefore balloon angioplasty may be an excellent choice for this patients’ group.

In this study, immediate results after balloon angioplasty showed a significant decrease in COA gradient after high pressure and low-pressure balloons, but high-pressure balloons showed more decrease in coarctation gradient.

Management of COA is indicated when the gradient during cardiac catheterization is more than 20 mmHg [5, 16]. In the present study, all the infants had a peak gradient of more than 20 mm Hg. That gradient decreased to below 20 mmHg just after balloon angioplasty. In a study by Oswal et al. (n = 44 infants), the mean gradient was decreased from 48.05 ± 15.26 mm Hg to 10.97 ± 5.8 mm Hg and showed a successful immediate result after the balloon angioplasty of COA [17]. Reduction in peak-to-peak pressure gradients across the COA, a significant increase in COA diameter by angiography, improved heart failure, and decreased hypertension following balloon angioplasty of COA reported in some studies [18–24].

In this study, 21% of infants needed reintervention. Other studies have reported a reintervention rate between 6 to 53% for recoarctation of the aorta. There was no statistically significant difference between the patients who need reintervention and others in the age of first balloon angioplasty, the stenotic part's diameter, pressure gradient before the first balloon, and pressure gradient after first balloon angioplasty. Also, other studies showed no difference between the two groups. The recoarctation rates were related to age at angioplasty than to the route, which the procedure was performed, and recoarctation was more in the neonate (< 30 days) [25].

In our study, the pressure gradient of COA decreased more in the high-pressure balloon group, and the rate of recoarctation was significantly lower in this group. For balloon angioplasty of COA, low profile balloons like Tyshak II balloons, which may be introduced through 4-French sheaths or Mini-Tyshack and can be entered via 3-French sheaths should be used. But a new high-pressure balloon for peripheral angioplasty has a low profile and can be entered via 5-Frech sheaths that can be used in infants [19].

Similar studies in all age groups reported the effect of balloon angioplasty immediately after the procedure. A low-pressure balloon is recommended for infants with a small body, particularly in cases with native CoA. In recoarctation cases, the site of ​​aortic tissue usually undergoes some degree of fibrosis. Thus, a low-pressure balloon may result in inconsistent results. However, the infant is usually provided with sufficient time to grow for stenting at an elder age [7]. In the present study, it was observed that the angioplasty balloon-type had no effects on ascending and descending aortic systolic pressure gradients in infants with native CoA, and the immediate results were similar after angioplasty with both types. Despite this finding, there was a difference between the two balloon types in terms of recoarctation, aneurysm, and mortality remains unclear.

In another study by our team, the rate of recoarctation was reported at four percent in children > 1 year of age [26], which is less than that the result of the present study (21.4%) and a rate of 7–30% reported [27]. Regarding the higher incidence of recoarctation in neonates than in elderly patients following balloon angioplasty, it may be explained that despite the rapid control of rupture and elongation in the intima and media with the balloon, the residual abnormal ductal tissue results in elastic recoil at the coarctation site. Besides, smooth muscle proliferation induces intimal hyperplasia, and the above two cases predispose the patient to recoarctation [28]. Some evidence by Atalay et al. presented that histologic findings such as neointimal proliferation, aortic intimal fibrosis in the arterial root, and ductal residual tissue debris together with a high-pressure gradient before balloon angioplasty can lead to an increased chance of recoarctation [29].

In this study, all age groups had similar immediate responses to decreased gradient, but age groups of < 1 month had more recoarctation than 1–3 months and 3–12 months. Similarly, Sen et al. reported that their studied parameters had no effects on recoarctation, and only the presence of residual coarctation in pre-discharge echocardiography was an independent predicting parameter [28].

In this study, the post-balloon angioplasty aneurysm rate was 7.2%, which varies widely (5–43%) in different studies. This discrepancy can be related to technical differences, duration of the follow-up period, lack of systematic imaging during follow-up in many studies, and differences in the aneurysm detection criteria [30, 31]. It is noteworthy that the extent and mechanism of aneurysm formation in CoA may also be explained by elastic fibers' rupture [32]. One aneurysm formation was in the low-pressure group and two of them were in the high-pressure group. Besides, all aneurysm formation was in group 2 of arch anatomy with hypoplastic isthmus that can be explained by dog bone shape formation during balloon angioplasty in this patients’ group.

It is still challenging to select the surgical technique or balloon angioplasty because most previous studies were retrospective and single-centered and evaluated the method that selected by health centers and surgeons. In most studies, there is a short follow-up period for the patients and limited data on mortality rates, and both methods are associated with the possibility of aortic rupture, aneurysm, recoarctation, other complications, and even death [13]. However, the evidence suggests several benefits for balloon angioplasty compared to surgery in neonates, especially those aged less than three months, and there is a considerable mortality rate in neonatal surgical technique. Furthermore, surgery is very challenging in recoarctation and it is associated with high morbidity and mortality, particularly in neonates, compared to native CoA surgery [8, 9], with a reported mortality rate of 0.7% [32]. One of the disadvantage of balloon angioplasty is higher risk of recoarctation compared to surgery; however, angioplasty or stenting is the choice technique, and surgery is avoided in the cases with recoarctation. Although the rate of the aneurysm is relatively higher in balloon angioplasty, most patients undergo follow-up and conservative treatment and do not experience surgery [29]. Overall, balloon angioplasty outcomes seem to be close to surgical results in infants < 3 months of age in terms of treatment options, but with fewer complications, shorter intubation duration, and more extended hospital stay [33]. Therefore, balloon angioplasty is effective in neonates and has advantages over surgery. However, selective treatment depends on the patient's age and clinical status, the stenosis's anatomy, and its adjacent structures [11, 34].

The present study suffers from some limitations, and small sample size to evaluate all related complications, and a lack of long-term follow-up.

## Conclusion

Generrally, there was no statistically significant difference between a high-pressure balloon and a low-pressure balloon to decrease the COA pressure gradient. The recoarctation rate was lower in the high-pressure balloon. The infant with discrete COA had a better response to the balloon with more decrease in gradient and lower recoarctation rate. Therefore, the stenotic segment anatomy needs to be considered in the selection of treatment methods.

## Data Availability

We state that the data used and/or analyzed during the current study are available from the corresponding author on reasonable request. Data sharing is applicable to this article and datasets were generated and analyzed during the current study and data sharing is allowed.
